# *Metschnikowia pulcherrima* and *Lachancea thermotolerans* Killer Toxins: Contribution to Must Bioprotection

**DOI:** 10.3390/foods14091462

**Published:** 2025-04-23

**Authors:** Fatima El Dana, Vanessa David, Mohammad Ali Hallal, Raphaëlle Tourdot-Maréchal, Salem Hayar, Marie-Charlotte Colosio, Hervé Alexandre

**Affiliations:** 1 UMR Procédés Alimentaires et Microbiologiques, Université de Bourgogne Europe, L’Institut Agro Dijon, INRAE, Laboratoire AFIM-IUVV, 21000 Dijon, France; fatima.eldana@gmail.com (F.E.D.); vanessa.vaizant@u-bourgogne.fr (V.D.); raphaele.tourdot-marechal@u-bourgogne.fr (R.T.-M.); 2 Doctoral School of Science and Technology, Platform for Research and Analysis in Environmental Science (EDST-PRASE), Lebanese University, Rafik Hariri Campus, Hadat-Baabda 1003, Lebanon; hayarsalem@gmail.com; 3 Department of Plant Protection, Faculty of Agronomy, Lebanese University, Dekwaneh-Matn 90775, Lebanon; mohammadali.hallal@st.ul.edu.lb; 4 Institut Français de la Vigne et du Vin (IFV), 44120 Nantes, France; marie-charlotte.colosio@vignevin.com

**Keywords:** bioprotection, killer toxin, *Metschnikowia pulcherrima*, Lachancea thermotolerans, metabolite analyses, *Brettanomyces bruxellensis*, *Hanseniaspora uvarum*, enology

## Abstract

The spoilage of wine caused by *Brettanomyces bruxellensis* and *Hanseniaspora uvarum* poses a significant challenge for winemakers, necessitating the development of effective and reliable strategies to control the growth of these yeasts, such as grape must bioprotection. Despite evidence that certain microorganisms can inhibit the growth of *Brettanomyces bruxellensis* and *Hanseniaspora uvarum*, the specific mechanisms driving this inhibition remain unclear. The primary objective of this study is to elucidate the underlying mechanisms responsible for this inhibitory effect. We analyzed one *Metschnikowia pulcherrima* (Mp2) and two *Lachancea thermotolerans* (Lt29 and Lt45) strains, all of which demonstrated significant killing and inhibitory effects on *Brettanomyces bruxellensis* (B1 and B250) and *Hanseniaspora uvarum* (Hu3137) in synthetic must at pH 3.5 and 22 °C. The effectiveness of these two strains exhibited varying inhibition kinetics. The strains were monitored for growth and metabolite production (L-lactic acid, ethanol, and acetic acid) in both single and co-cultures. The low levels of these metabolites did not account for the observed bioprotective effect, indicating a different mechanism at play, especially given the different growth profiles observed with added L-lactic acid and ethanol compared to direct bioprotectant addition. Following the production, purification, and quantification of killer toxins, different concentrations of toxins were tested, showing that the semi-purified Mp2Kt, Lt29Kt, and Lt45Kt toxins controlled the growth of both spoilage yeasts in a dose-dependent manner. These bioprotectant strains also showed compatibility with *Saccharomyces cerevisiae* in co-cultures, suggesting their potential use alongside commercial starter cultures.

## 1. Introduction

*Brettanomyces bruxellensis* and *Hanseniaspora uvarum* are considered major wine spoilage contributor yeasts. Their presence and proliferation in wine are primarily controlled through the application of sulfur dioxide (SO_2_) from the beginning of winemaking [1,2]. SO_2_ is regarded as a crucial tool for winemakers because of its affordability and its dual antioxidant and antimicrobial properties, which are effective against a broad spectrum of microorganisms [3]. However, besides reports that such yeast species have developed tolerance to wide range levels of SO_2_, sulfites can cause allergic reactions in sensitive consumers [4]. This has resulted in a growing societal demand for wines free or with minimal quantities of SO_2_ [5]. To address these challenges, the concept of bioprotection has emerged as a promising alternative method [6].

Bioprotection is a relatively recent term and an emerging concept across wine industries. It refers to natural or controlled agents, including microorganisms, enzymes, or biological antimicrobial compounds employed to prevent or suppress the proliferation of harmful pathogens without impairing the sensory properties of the product [7,8,9,10]. The primary objectives of bioprotection are to safeguard must from spoilage at early stages, control oxidation, and prevent off-flavors through establishing interactions between the bioprotective strain and the native microbiota, thereby leading to the inhibition of indigenous yeast populations [7,11,12]. This approach aligns with natural winemaking practices, offering a more sustainable alternative to SO_2_ for preserving wine quality especially during the pre-fermentation stages of winemaking.

Different interaction mechanisms can be involved in bioprotection, each of which has been identified recently as being yeast species-dependent. The interactions involved can occur through both indirect and direct actions [7,13,14]. Indirect interactions include competition for essential nutrients, space, oxygen consumption [7,14,15,16,17,18,19], the modification of the medium’s acidity, and the release of VOCs (e.g., 2-phenylethanol produced by *Pichia kudriavzevii*) [20,21]. We can add the production of antimicrobial compounds such as killer toxins, pulcherriminic acid, ethanol, acetic acid, etc. [13,22,23,24]. On the other hand, direct interactions include cell–cell contact, which can lead to physical interference or signaling processes that directly affect the growth and behavior of other microorganisms [7,13]. However, some of these are hypotheses and have not been demonstrated as mechanisms for bioprotection.

A recent phenotype has emerged involving the secretion of killer toxins, which may play a key role in bioprotection and can be harnessed to control the growth of unwanted microorganisms in wine [25]. Killer toxins are typically proteinaceous antimicrobial compounds that bind to specific receptors on the surface of target microorganisms, leading to their death through a targeted mode of action. Non-*Saccharomyces* yeasts such as *Kluyveromyces wickerhamii*, *Pichia membranifaciens*, *Metschnikowia pulcherrima*, and *Wickerhamomyces anomala* have been identified as producers of killer toxins with antimicrobial activity against *Brettanomyces* and *Hanseniaspora* genera [7,26,27,28,29].

Numerous co-culture experiments have been carried out, demonstrating the inhibitory effect of one species on another. The authors have often made the hypothesis that this inhibition is linked to the production of killer toxins based on the fact that on agar medium the species had a killer effect. However, these experiments did not demonstrate the real involvement of killer toxins on inhibition in a wine environment and did not take into account the involvement of other interaction mechanisms. For example, in the study by Santos et al. [30], the inhibition of *Brettanomyces bruxellensis* by *Ustilago maydis* in grape juice medium was attributed to the killer effect determined on agar medium without taking into account the existence of other inhibition mechanisms. Furthermore, in many studies regarding the killer toxin effect, the experimental conditions were very far from winemaking conditions.

The inhibition of yeasts by killer toxins secreted by *Kluyveromyces wickerhamii* (KwKt DBVPG 6077) and *Pichia anomala* (PiKt, DBVPG 3003) was demonstrated [26] in YEPD and Sangiovese wine medium. However, KwKt and PiKt were produced before in YEPD instead of wine [26]. Incubation at 25 °C and 35 °C at a pH of 4.4 showed the highest inhibition diameter on agar plates for KwKt and PiKt, respectively. As stated, a significant reduction in the population of sensitive yeast cells in wine was observed [26]. The production of killer toxin from the same yeast species was also achieved in [31] using similar parameters but in Yeast Malt Peptone (YMP) medium and Yeast Nitrogen Base (YNB) medium and showed a similar inhibitory effect. In addition, a recent study illustrated the secretion and effect of a killer toxin of 7.4 KDa by a *Metschnikowia pulcherrima* strain (TB26) isolated from grapevine [32].

Moreover, the zymocidal effect of KpKt (from *Kluyveromyces phaffi* DBVPG 6706) was assessed in pasteurized Trebbiano toscano grape must (microfermentation) [29,33,34]. However, the toxins were not produced; instead, the strain was used in a free and immobilized state against *Hanseniaspora uvarum* cultivated in YPD at 25 °C [29,34]. Similarly, the release and efficacy of antimicrobial compounds CpKt1 and CpKt2 (50 KDa) produced by *Candida pyralidae* in YPD broth [35,36] exerted killing activity mainly on Wort Yeast Extract (WYE) agar plates after 36 h [35]. However, this study revealed that several *Brettanomyces bruxellensis* strains exhibited resistance to KwKt and CpKt when grown on YPD agar. Additionally, although *Tetrapisispora phaffii* (CBS 4417) has been reported to inhibit *Hanseniaspora uvarum* [29,33], recent findings [35] indicated that they did not inhibit the growth of any *Hanseniaspora uvarum* or *Brettanomyces bruxellensis* strains tested in the study. This discrepancy may be due to differences in the screening media, suggesting that killer activity depends on both the strains involved and the medium used, warranting further investigation.

Previous studies on the production of killer toxins have primarily utilized laboratory media at high pH values (~4.5), conditions that may not fully represent the complexities of real oenological environments [33,37,38,39,40]. The behavior of killer yeast strains could differ when exposed to the actual composition and lower pH value (~3.5) of wine. Additionally, many studies have assessed toxin efficacy on agar plates, which may not accurately simulate wine-like media, potentially leading to discrepancies in the results. However, only a few studies have evaluated the efficacy of extracted killer toxins against *Brettanomyces bruxellensis* and *Hanseniaspora uvarum* in grape must and wine at 25 °C. These investigations have documented the effectiveness of these toxins as bioprotective agents [37,41].

This present study evaluates the effectiveness of one *Metschnikowia pulcherrima* strain and two *Lachancea thermotolerans* strains in synthetic must, artificially contaminated and adjusted to a pH of 3.5 at 22 °C to replicate fermentation conditions. Two spoilage yeast species, *Brettanomyces bruxellensis* and *Hanseniaspora uvarum*, were tested. They simulate the natural occurrence of indigenous yeasts that can vary based on different environmental conditions. To understand the physiological mechanisms underlying bioprotective effectiveness, the impact of metabolites on the inhibition of these spoilage strains was analyzed. The role of killer toxins as a mechanism of bioprotection was also investigated.

## 2. Materials and Methods

### 2.1. Yeast Strains

Bioprotective *non-Saccharomyces* yeasts isolated, identified, and characterized in the laboratory—*Starmerella bacillaris* (formerly known as *Candida stellata*), *Lachancea thermotolerans*, and *Pichia kudriavzevii*—were isolated, identified, and characterized from 12 grape varieties collected during an autochthonous yeast isolation campaign at the Kefraya vineyard in Northern Lebanon. All target-sensitive yeast strains of *Brettanomyces bruxellensis* coded B1, B250, B3 (NL6293), and B7 (NL6297) and of *Hanseniaspora uvarum* coded Hu3137 came from the Yeast Collection of the French institute of vine and wine (Institut Français de la Vigne et du Vin (IFV), Nantes, France) and the University Institute of Vine and Wine Jules Guyot (Institut de la Vigne et du Vin de Université Bourgogne Europe (IUVV), Dijon, France), respectively. Additionally, a commercial *Metschnikowia pulcherrima* strain “Mp2”, used as a bioprotectant, and the commercial *Saccharomyces cerevisiae* strain VL2 were provided by IFV, while three other *Saccharomyces cerevisiae* strains, S340 (174 COEB), S342 (NCYC 738), and S334 (NCYC 1006), were purchased (collection strains) or given.

### 2.2. Growth Media

All the yeast strains were pre-cultivated at 22 °C overnight in 40 mL of YPD broth containing 10 g/L yeast extract, 20 g/L peptone, 20 g/L glucose, and 18 g/L agar, with the addition of 0.2 g/L of chloramphenicol (Sigma-Aldrich, Saint-Quantin-Fallavier, France), and buffered at pH 3.5 using 0.1 M hydrochloric acid/citric acid. For the *Brettanomyces bruxellensis* strains, the incubation period was extended to 72 h due to their slow growth. All the yeasts were stored in 20% (*v*/*v*) glycerol (Sigma-Aldrich, Saint-Quantin-Fallavier, France) with YPD broth at −80 °C.

For the killer activity assay on agar, YPD-BM (YPD supplemented with 30 g/L methylene blue, 20 g/L agar) was used, whereas for the killing potentiality assay in liquid medium under oenological conditions and for killer toxin production, synthetic must medium SM adjusted to 3.5 was used in Erlenmeyer flasks covered with carded cotton, each containing 100 mL of sterile synthetic must MS300 (referred to as SM), and the composition was previously described by Evers et al. [42].

### 2.3. Killer Assay of Bioprotective Yeast Species

The killer activity of a panel of autochthonous yeasts *Starmerella bacillaris*, *Pichia kudriavzevii*, *Lachancea thermotolerans*, and commercial Mp2 strain was examined against 4 sensitive *Brettanomyces bruxellensis* strains, B1, B250, B3, and B7, and 1 *Hanseniaspora uvarum* Hu3137 with the diffusion plate assay. Sensitive strains at final concentrations of approximately 10^6^ cells/mL were uniformly mixed with 20 mL of YPD-MB, buffered to pH 3.5. The agar mixture, maintained at 45 °C in a water bath, was immediately poured into sterile petri dishes [37,41,43,44]. Killer yeast strains, prepared at a concentration of 10^6^ cells/mL, were spotted onto the agar surface. Plates were incubated at 22 °C for 72 h, and killer activity was identified by clear zones of inhibition surrounding the spot. Killer activity (KA) was measured in arbitrary units (AU) per mL and calculated using the inhibition zone’s diameter in millimeters, where 1 AU represents the quantity of toxin needed to produce a clear inhibition zone with a diameter of 13 mm. All trials were carried out in triplicate [26,37,41,44,45].

### 2.4. Assessing Killer Activity in Co-Cultures of Bioprotectant Against Saccharomyces cerevisiae

The impact of the selected bioprotectant yeasts, Lt 29129 (Lt29), Lt 28645 (Lt45), and Mp2 (see Section 3.1), on an alcoholic performance stack was tested against a panel of commercial *Saccharomyces cerevisiae* strains, VL2, S342, S340, and S334, by the diffusion plate assay. Plates were seeded with *Saccharomyces cerevisiae* strains at a final concentration of 10^6^ CFU/mL on MB-YPD adjusted to pH 3.5. An exponential-phase culture of bioprotectant yeast strains was spotted after 48 h of incubation at 22 °C. Strains were classified as sensitive if the spot was surrounded by a clear inhibition zone. The assay was performed in triplicate, and the diameter of the inhibition zone was measured using a caliper. Killer activity (KA) was expressed in arbitrary units (AU) per mL.

### 2.5. Monitoring Bioprotectant Killing Activity in Synthetic Must

The bioprotectant and sensitive strains utilized in this study are detailed in Table 1. The effect of bioprotectants on spoilage yeasts was assessed using the conditions that gave the best results with different temperatures, pH values, and population densities. Pre-cultures of all yeast strains were prepared by incubating in 40 mL synthetic must at 22 °C with continuous agitation on a rotator overnight, except for *Brettanomyces bruxellensis*, which was incubated for 72 h. For single cultures, each strain was inoculated independently at an initial concentration of 1·10^6^ viable cells/mL for bioprotectant yeasts *Metschnikowia pulcherrima* (Mp2) and *L. thermotolerance* (Lt29 and Lt45) and 1·10^4^ viable cells/mL for the sensitive strains *Brettanomyces bruxellensis* (B1 and B250) and *Hanseniaspora uvarum* (Hu3137). These single cultures served as controls. For the co-culture experiments, each bioprotectant yeast of concentration 1·10^6^ cells/mL was co-inoculated with 1·10^4^ cells/mL *Brettanomyces bruxellensis* or *Hanseniaspora uvarum* strains (to replicate the possible occurrence of this yeast during the pre-fermentative stage) [46], at T_0_ of incubation, as outlined in Table 1. Cultures were maintained at 22 °C, and samples of 100 μL were collected from each flask (mono- and co-cultures) at 0, 1, 2, 3, 6, 7, and 9 days post-inoculation for cell enumeration and then stored at −20 °C for subsequent chemical analyses.

The enumeration of *Lachancea thermotolerans* and *Metschnikowia pulcherrima* strains was conducted on YPD and WL Oxoïd CM0309 (Oxoïd, Hampshire, UK) medium, to which 0.2 g/L chloramphenicol was added, where they formed red colonies as a result of pulcherrimin production [47]. Populations of *Hanseniaspora uvarum* and *Brettanomyces bruxellensis* cells were enumerated by counting on a modified ITV selective medium (20 g/L glucose, 10 g/L yeast extract, 20 g/L tryptone, 0.1 g/L para-coumaric acid, 0.1 g/L ferulic acid, 0.03 g/L green bromocresol, 0.2 g/L chloramphenicol, and 20 g/L agar, adjusted to pH 3.5, supplemented with 0.006% (*v*/*v*) cycloheximide), after incubation for 48 h and 72 h, respectively, at 22 °C. All experiments were conducted in triplicate.

### 2.6. Partial Production and Purification of Killer Toxins

Single yeast cultures (Mp2, Lt29, and Lt45) were pre-adapted in synthetic must (SM) of pH 3.5 at 22 °C for 18 h. Subsequently, 10% (*v*/*v*) of the active culture was inoculated in 300 mL SM (pH 3.5) in 500 mL Erlenmeyer flasks and incubated at 22 °C for 24 h with shaking at 150 rpm. After the observation of a visible turbidity in the single cultures, yeast cells were separated by centrifugation at 4100 rpm for 30 min at 4 °C [32,48]. The supernatant was collected and added to 96% ice-cold ethanol at a ratio of 2:1 (*v*/*v*). The mixture was allowed to stand at 4 °C overnight and subsequently centrifuged at 12,000 rpm for 20 min at 4 °C. The precipitate containing the killer toxin proteins was separated from the liquid phase, dissolved in 0.1 M sodium phosphate buffer (pH 7.0), and stored at 4 °C for further use [32,48].

The purified toxins Lt29Kt, Lt45Kt, and Mp2Kt were tested for antimicrobial activity using agar well diffusion and in SM through cell enumeration methods. Approximately 3–4 mL of pure toxin was obtained from 300 mL of each bioprotectant culture. The mode of action and activity of the purified killer toxins was assessed on sensitive *Hanseniaspora uvarum* and *Brettanomyces bruxellensis* strains to evaluate their inhibitory potential on agar medium and in synthetic must. Based on a prior study by Agarbati et al. [31], highlighting the efficiency of three toxins at a concentration range within 0.1–1.0 mg/mL towards *Brettanomyces bruxellensis*, two concentrations, 0.185 mg/mL and 0.5 mg/mL, were tested.

### 2.7. Quantification of Protein Concentration

Following partial purification processes, the presence of protein produced by bioprotectant yeasts (Mp2, Lt29, and Lt45) was assessed using the Bradford assay (1976). The Bradford assay is a colorimetric method based on protein–dye binding. A standard curve was established using bovine serum albumin (BSA) standards (0.1–1.0 mg/mL) mixed with Bradford reagent. Absorbance was measured at 595 nm with a UV–Vis spectrophotometer (Genesys 50, Thermo Fisher Scientific Inc., Waltham, MA, USA), and protein concentrations of the yeast extracts were automatically calculated from the standard curve and expressed in mg/mL.

### 2.8. Evaluation of Toxin’s Killing Activity

Killer activity (KA) was determined by the diffusion plate assay and expressed as arbitrary units (AU)/mL. The plates were seeded with *Brettanomyces bruxellensis* (B1 and B250) and *Hanseniaspora uvarum* (Hu3137) at a final concentration of 1.10^4^ cells/mL on solidified YPD-MB agar, pH 3.5. Then, a 5 mm halo was formed in the agar, where two concentrations 0.185 mg/mL and 0.500 mg/mL of partiality purified killer toxin containing a fraction of each of the bioprotectants of Mp2, Lt29, and Lt45 were seeded inside it. The plates were incubated at 22 °C for 24–48 h, after which the diameter of the clear inhibition zones was measured using a caliper. A regression analysis performed on Excel established a linear relationship between the diameter and the logarithm of the AU of toxins, following the empirical formula: $KA(AU/mL)=10^{(D+5.64)/6.64}$, where D is the inhibition zone diameter (millimeters) and 1 AU corresponds to the quantity of toxin capable of creating a 1 mm inhibition zone [38].

### 2.9. Assessment of Killer Toxin’s Inhibitory Potentiality in Synthetic Must

This protocol was designed to evaluate the killing and inhibitory activity of partially purified killer toxins produced by *Metschnikowia pulcherrima* and *Lachancea thermotolerans* in synthetic must, simulating the nutritional composition of wine. Single cultures of each sensitive yeast strain were inoculated at an initial concentration of 1·10^4^ cells/mL in 50 mL glass bottles containing 40 mL of synthetic must adjusted to pH 3.5. Each culture was treated with two different concentrations of purified killer toxins (Mp2Kt, Lt29Kt, and Lt45Kt), specifically 0.185 and 0.500 mg/mL, and incubated at 22 °C. At designated intervals (0 h, 5 h, 24 h, and 72 h), aliquots were collected, where viable cell counts were performed in triplicate using YPD agar plates and D_600_ was measured. The plates were incubated at 22 °C, and colony counts were recorded to assess population reduction, expressed as CFU/mL over time. Killing and inhibitory activity was expressed as the absence of and reduction in sensitive strain populations, respectively, of a culture compared with growth control.

### 2.10. Evaluation of Metabolite Production as Mode of Action

#### 2.10.1. Lactic Acid and Acetic Acid Analysis

Throughout the incubation period of the monocultures and co-cultures in glass bottles containing 40 mL synthetic must (pH 3.5), samples were taken and analyzed daily at 22 °C, for the entire run time of the experiment (9 days), in conjunction with the population monitoring of the bioprotectant and sensitive yeast strains (Section 2.5). A volume of 200 μL of each culture medium was sampled and centrifuged at 10,000 rpm for 5 min, and the supernatant was frozen at −20 °C for metabolic by-product analysis. L-lactic acid and acetic acid concentrations were monitored by an automated Y15 sequential analyzer (BioSystem, Muttenz, Switzerland), using an L-lactic acid RA and RB (ref:12802) reagent kit and an acetic acid RA and RB reagent kit, respectively, prior to controlling with red wine control (Ref: 2822) (BioSystem, Muttenz, Switzerland).

#### 2.10.2. Ethanol, Malic Acid, Acetic Acid, and Total Acidity Analysis

Measurements of ethanol, malic acid, acetic acid, and total acidity (TA) were determined by FT-IR spectroscopy with a OenoFoss™ analyzer (Ecoparc de Nanterre, Nanterre, France) throughout the 9 days of incubation at 22 °C of each of the monocultures and co-cultures in synthetic must at pH 3.5. The OenoFoss™ analyzer (FOSS, Nils Foss Allé 1, DK-3400 Hilleroed, Denmark) was calibrated using a fresh wine sample (Cabernet Sauvignon, Roche Mazet, Pays-d’oc, France) until accurate calibration was achieved. A total of 500 μL of each culture was taken and centrifuged at 7000 rpm for 10 min, and then 200 μL of the supernatant was inserted in the opening lens of the machine using the Must Under Fermentation (FMS) test option.

### 2.11. Statistical Analysis

Growth parameters and killer toxins’ efficiencies were analyzed using one-way analysis of variance (ANOVA). Levene’s test was applied to assess the homogeneity of variances. For comparisons between two groups where variances were not equal, Welch’s *t*-test was used. The significance level was set at an α of 0.05. Metabolite production and relative intensities were analyzed using Welch’s *t*-test when comparing two groups (*n* = 2) and an ANOVA followed by Tukey’s post hoc test for more than two groups (*n* = 3), maintaining a 5% significance level. The examination of cell growth, maximum growth rate (µmax h^−1^), and graphical outputs, along with other statistical analyses, was conducted using IBM SPSS Statistics (version 22).

## 3. Results and Discussion

### 3.1. Screening for Potential Yeast Strains with Killer Activity Against Brettanomyces bruxellensis and Hanseniaspora uvarum

The assessment of the activity spectrum of the yeast species and strains studied represented an essential initial step toward the practical utilization of its killer toxin for managing spoilage yeasts in winemaking processes. Twenty autochthonous and one commercial killer yeast strains from four species—*Starmerella bacillaris*, *Lachancea thermotolerans*, *Pichia kudriavzevii*, and *Metschnikowia pulcherrima*, respectively—were assessed for their inhibitory effects in agar on four *Brettanomyces bruxellensis* strains (B1, B250, B3, and B7) and one *Hanseniaspora uvarum* strain (Hu3137). The 20 autochthonous tester strains were originally isolated from wine grape sources.

The symbols in the fourth column describe how killer toxins affected the four *Brettanomyces bruxellensis* strains, B1, B250, B3, and B7, and one *Hanseniaspora uvarum* strain, Hu3137, on YPD-BM agar. Legends: ++ strong killer effect (diameter of halo 10–13mm); + apparent killer effect (diameter of halo between 8 and 9 mm); +/− mild killer effect (diameter of halo < 8 mm and not consistent); − no killer effect. Among these species, *Pichia kudriavzevii* displayed either negligible or only mild inhibitory effects against the *Brettanomyces bruxellensis* strains (Table 2). However, most of the *Brettanomyces bruxellensis* strains excluding B7 were apparently sensitive to the two *Starmerella bacillaris* strains. In contrast, as detailed in Table 3, *Lachancea thermotolerans* exhibited strain-specific variations in activity, ranging from strong, apparent, to mild killing potential, when tested at an equal population density of 1·10^6^ CFU/mL and incubated at 22 °C. Notably, seven strains of *Lachancea thermotolerans* (47%)—Lt 28645, Lt 28655, NL 29128, Lt 29129, Lt 29130, Lt 29143, and Lt 29147—demonstrated consistent activity against all the *Brettanomyces bruxellensis* strains. Among these, only Lt 29129 and Lt 28645 demonstrated strong activity against *Hu*3137; the activity of the remaining strains against *Hanseniaspora uvarum* was not determined (Nd). *Metschnikowia pulcherrimin* (Mp2) showed strong to apparent activity against all the sensitive strains (Table 2).

**Figure 1:**
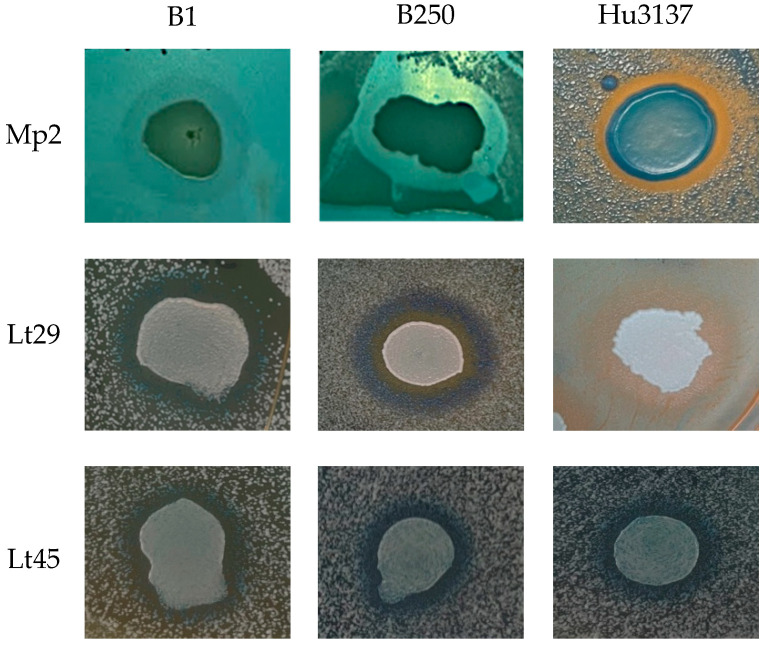
Agar plate assay for killing activity. Bioprotectant strains *Metschnikowia pulcherrima* (Mp2) and *Lachancea* thermotolerance (Lt29 and Lt45) cells inoculated on top of YPD-MB medium (pH 3.5) previously spread with a lawn of sensitive strains *Brettanomyces bruxellensis* (B1 and B25) and *Hanseniaspora uvarum* (Hu3137) and incubated at 22 °C. Final concentration 10^6^ CFU/mL for both killer and sensitive strains was maintained. Halos represent death zone, indicated by precipitate of methylene blue.

To further investigate the effectiveness of these killer yeasts in liquid medium and their ability to maintain activity beyond agar-based assays, three representative strains were selected for additional study: *Lachancea thermotolerans* (Lt 29129 as Lt29 and Lt 28645 as Lt45) and *Metschnikowia pulcherrima* (Mp2), which exhibited broad-spectrum activity against all sensitive *Brettanomyces bruxellensis* and *Hanseniaspora uvarum* strains (Figure 1).

These findings are in line with previous studies highlighting the efficacy of killer yeast strains in suppressing spoilage yeasts on agar [26,35,41]. However, it is important to note that these tests were conducted at a pH range of 4.2 to 4.5, which does not accurately reflect the pH conditions during winemaking.

### 3.2. Co-Culture of Bioprotectant Yeast Species with Saccharomyces cerevisiae

Prior to evaluating the efficiency of killer yeasts applied as bioprotectant agents against spoilage yeasts in liquid medium, it was crucial to assess their impact on *Saccharomyces cerevisiae* strains used for alcoholic fermentation and the latter’s ability to inhibit the bioprotectants’ growth. All three bioprotectant strains (Mp2, Lt29, and Lt45) demonstrated resistance to the four *Saccharomyces cerevisiae* strains tested (Table 3). This finding contrasts with previous studies reporting the potentiality of *Saccharomyces cerevisiae* to inhibit the growth of some strains belonging to *Lachancea thermotolerans* and *Metschnikowia pulcherrima* upon co-inoculation [49,50,51,52,53]. Notably, Lt28645 exhibited killing activity against two sensitive strains, S334 and VL2 (Table 3), consistent with earlier research reporting the ability of *Lachancea thermotolerans* to suppress *Saccharomyces cerevisiae* [50]. This provides a basis for selecting Lt28645 to further investigate its mechanisms of action using a well-characterized biological model. In contrast, as shown in Table 3, Mp2 and Lt29129 showed no inhibitory activity against the entire set of *Saccharomyces cerevisiae* strains, while maintaining resistance to potential antagonism by these strains. This dual property makes Mp2 and Lt29129 promising bioprotectant candidates for further study in wine production, as it aids in selecting compatible killer yeast strains without adverse effects on fermentation performance.

### 3.3. Bioprotective Potentiality in Synthetic Must

Among the potential mechanisms that have been studied is competition for nitrogen sources and the production of pulcherriminic acid [46,54]. As far as we know, no study has previously focused on the role of killer toxins in yeast interaction mechanisms during must bioprotection.

**Figure 2:**
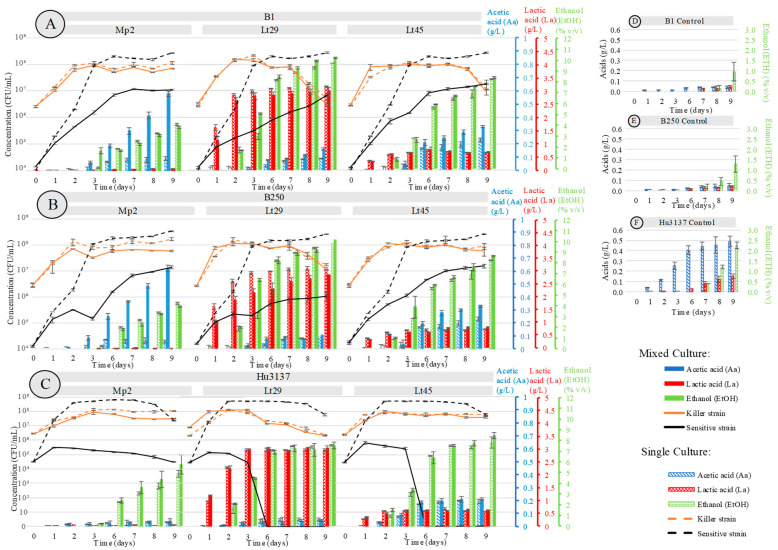
Growth curves at 22 °C of Hanseniaspora and Brettanomyces in single culture (black dotted line) and co-culture (black solid line) in presence of (**A**) B1 in co-culture, (**B**) B250 in co-culture, and (**C**) Hu3137 in co-culture at initial concentration 10^4^ and 10^6^ CFU/mL, respectively, throughout fermentation period (9 days). (**D**) B1 control, (**E**) B250 control, (**F**) Hu3137 control. Profiles of bioprotectant strains in single culture (orange dotted line) and mixed culture (black solid line) when inoculated with spoilage, at initial concentration of 10^6^ CFU/mL. Metabolite production by Mp2, Lt29, and Lt45 in single culture (dashed bars) and in co-culture (solid bars) with B1, B250, and Hu3137; 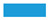 acetic acid (Ac); 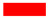 L-lactic acid (La); 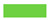 ethanol (EtOH).

Yeast growth profile curves during cultivation in synthetic must (pH 3.5) with spoilage yeasts in single culture and co-culture with the selected bioprotectant yeasts are presented in Figure 2. The profiles were monitored over the course of the fermentation period, which lasted 9 days. For the co-culture, a single contamination level of an initial concentration 1·10^4^ CFU/mL of *Brettanomyces bruxellensis* or *Hanseniaspora uvarum* alone was conducted. Each bioprotectant strain, including *Metschnikowia pulcherrima* (Mp2) and *Lachancea thermotolerans* (*Lt*29 and *Lt*45), was inoculated at a biomass of 1·10^6^ CFU/mL. To assess the impact of antagonistic interactions on the growth dynamics of each bioprotectant/*Brettanomyces bruxellensis strains* and bioprotectant/*Hanseniaspora uvarum strain*, kinetic parameters were analyzed and compared between single and co-cultures for each initial concentration of *Brettanomyces bruxellensis* and *Hanseniaspora uvarum* strains. Single culture growth parameters served as control values to determine changes resulting from the interactions (Figure 2).

All three bioprotectant strains, *Metschnikowia pulcherrima* Mp2 and *Lachancea thermotolerans* Lt29 and Lt45, when co-cultured with *Brettanomyces bruxellensis* and *Hanseniaspora uvarum* at an inoculation level of 1.10^6^ CFU/mL each, exhibited growth parameters comparable to those observed in their respective single cultures (Figure 2A–C), with no significant difference (*p* > 0.05). Bioprotectant strains were not negatively impacted (population) by the interaction throughout the 9 days when co-cultured with B1, B250, and Hu3137 strains (Table 4). The comparison was performed statistically within multiple groups.

**Figure 3:**
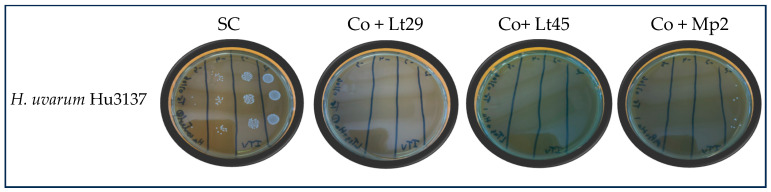
Killing efficiency of bioprotectant strains Lt29, Lt45, and Lt45 on *Hanseniaspora uvarum* in co-cultures mix in synthetic must pH 3.5 inoculated at 22 °C after 6 days of inoculation. *Hanseniaspora uvarum* cell viability was evaluated by viable plate count at 72 h of incubation on ITV agar. SC: Single culture of Hu3137 as control; Co: Co-culture; Co + Lt29: Hu3137 with Lt29; Co + Lt45: Hu3137 with Lt45; Co + Mp2: Hu3137 with Mp2. Each strain under same conditions was tested using same dilution (10^−2^, 10^−3^, 10^−4^, 10^−5^). Results reflect three technical replicates from three independent experiments.

Regarding their bioprotective effect, with an initial population of 1·10^4^ CFU/mL of *Hanseniaspora uvarum* Hu3137, strains Mp2, Lt29, and Lt45 demonstrated significant changes in the growth parameters of Hu3137. In co-cultures with Lt29 or Lt45, *Hanseniaspora uvarum* Hu3137 grew during the first 24 h of fermentation, reaching 1.10^5^ CFU/mL and 9·10^5^ CFU/mL, respectively, compared to the control value of 3·10^7^ CFU/mL, exhibiting different maximum specific growth rates (Figure 2C and Table 4). Subsequently, the *Hanseniaspora uvarum* Hu3137 population started to die off, and death was complete (fewer than 1 CFU/mL) within 3–6 days (Figure 3) of fermentation in the presence of both *Lachancea thermotolerans* bioprotectant strains, resulting in a statistically significant reduction in the growth of both spoilage yeasts compared to control treatments (*p* < 0.01), as shown in Figure 2C. The mechanism causing the premature death of *Hanseniaspora uvarum* in a synthetic must medium in the presence of the two *Lachancea thermotolerans* may be of a different nature. This could be linked to competition for nutrient sources. But this hypothesis can be ruled out. Indeed, the medium contained sufficient nutrients to reach a population of 5·10^8^ CFU/mL for the control culture of *Hanseniaspora uvarum* and 10^8^ CFU/mL for the control culture of *Lachancea thermotolerans*. In the co-culture, the maximum population reached was 10^5^ cells/mL, far from the maximum possible. Another possibility is the production of certain inhibitory metabolites such as lactic acid, for example, or ethanol [49,55,56]. Indeed, these two metabolites are known to be able to inhibit cell growth. However, the concentrations present during the death of *Hanseniaspora uvarum* (2% ethanol *v*/*v* and 2 g/L lactic acid) cannot be responsible for cell death (Figure 2C), especially for Lt45. Furthermore, both *Hanseniaspora uvarum* and *Brettanomyces bruxellensis* exhibited high ethanol tolerance, up to 9–12% and 14% *v*/*v*, respectively, as previously stated [57,58,59]. In these conditions, our results suggest that premature cell death could be linked to killer activity. For Lt29, a synergistic action of ethanol and lactic acid was possible since when *Hanseniaspora uvarum* started to die, the medium contained up to 4% ethanol and 3 g/L of lactic acid. However, we conducted an experiment in which *Hanseniaspora uvarum* growth was monitored in the presence of different concentrations of lactic acid and ethanol (Figure 2C), and it appeared that the synergistic action of these two metabolites did not lead to cell death as observed, but to a reduction in the maximum population (*p* < 0.05). This was expected since it has been previously demonstrated that *Hanseniaspora uvarum* exhibits high ethanol tolerance, up to 12% (*v*/*v*) [58,59]. These results suggest that cell growth arrest may be linked more to the presence of killer toxins, whereas the cell death observed after 6 days might be linked to the presence of ethanol. 

To our knowledge, this is the first time that the inhibition of *Hanseniaspora uvarum* by *Lachancea thermotolerans* has been reported. Indeed, experiments with co-cultures with *Lachancea thermotolerans* have been published [57,60], in which the authors did not describe any inhibition; however, the inoculation concentrations were far higher (10^6^ cells of each strain) compared to our ratio.

When *Hanseniaspora uvarum* Hu3137 was co-cultured with *Metschnikowia pulcherrima* Mp2, the final population of Hu3137 was significantly reduced, showing an approximate decrease of 3.5 log compared to its growth in single cultures (Figure 2C). The co-culture exerted a pronounced impact on the specific growth rate of Hu3137 (*p* < 0.05), as shown in Table 4. In contrast, in single cultures, *Hanseniaspora uvarum* Hu3137 exhibited a higher μmax value 0.204 ± 0.001 h^−1^ (Table 4) and reached a final population of 2·10^7^ CFU/mL (*p* < 0.05).

In the co-culture, *Metschnikowia pulcherrima* Mp2 demonstrated an inhibitory effect on *Hanseniaspora uvarum*, resulting in a significantly lower population throughout the entire fermentation period compared to single cultures. However, compared to *Lachancea thermotolerans*, no drastic cell death could be observed (*p* < 0.05) (Figure 2C). For the same reasons explained above, competition for nutrients could be ruled out as a mechanism explaining the inhibition of *Hanseniaspora uvarum* growth. Similarly, metabolite accumulation could not explain the inhibition since their concentrations (0.028 g/L for lactic acid, 0.1 % *v*/*v* for EtOH) in the medium when *Hanseniaspora uvarum* declines were not inhibitory [61]. For *Metschnikowia pulcherrima*, another potential inhibitory mechanism has been reported [46]. These authors proposed that the antimicrobial activity exerted by *Metschnikowia pulcherrima* against *Hanseniaspora guilliermondi* appears to be related to the diffusion of pulcherriminic acid as a precursor of pulcherrimin, which immobilizes the iron in the growth medium, thus reducing iron availability and inhibiting yeast, whose growth requires iron (Fe^3+^). However, in this present study, this mechanism did not seem to be involved. Indeed, the synthetic must was not supplied with iron. In these conditions, the hypothesis of inhibition due to killer toxins could also be put forward. Similar findings were reported in [24], where Mp2 was inoculated against a 50:50 mixed culture of *Hanseniaspora uvarum* (Hu3137) and *Hanseniaspora valbyensis* (ScS) at an initial concentration of 5·10^4^ CFU/mL, implemented at a lower temperature of 12 °C. These results highlighted the capacity of *Metschnikowia pulcherrima* as a bioprotective yeast capable of inhibiting spoilage *Hanseniaspora uvarum* species across a temperature range of 18–22 °C, conditions that closely mimic those encountered in winemaking environments.

Concerning the implantation of *Metschnikowia pulcherrima* Mp2 and two *Lachancea thermotolerance* Lt29 and Lt45 strains in mixed culture with the two sensitive *Brettanomyces bruxellensis strains* B1 and B250, all three of the bioprotectant co-cultures demonstrated typical growth dynamics, where all strains retained high cell viability during fermentation, as in single cultures (ex. single culture Lt29: 2.18·10^7^ CFU/mL, co-culture Lt29 + B250: 1·10^7^ CFU/mL) with no significant difference ( *p* > 0.05).

**Figure 4:**
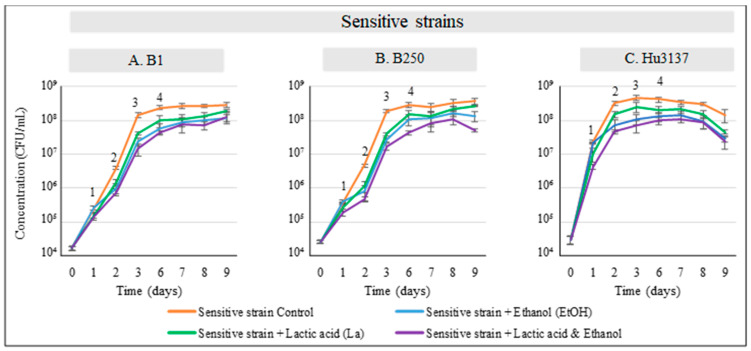
Synergism and sensitive yeast growth in synthetic must pH 3.5. Population growth of B1, B250, and Hu3137 was assessed through cell enumeration on agar in presence of increasing amounts of lactic acid and/or ethanol. (**—**) Sensitive control; (**—**) g/L lactic acid; (**—**) % (*v*/*v*) EtOH; (**—**) g/L lactic acid + % EtOH. (1) 1 mg/L lactic acid; (2) 1 g/L lactic acid + 3% EtOH; (3) 1 g/L lactic acid +2% EtOH; (4) 3% EtOH. Error bars displayed on line graphs indicate standard deviation from three replicates. (**A**) B1 single culture, (**B**) B250 single culture, (**C**) Hu3137 single culture.

In the co-culture of *Metschnikowia pulcherrima* Mp2 and *Brettanomyces bruxellensis* B1, a noticeable reduction in B1 biomass was observed within 48 h of inoculation, accompanied by lower specific growth rates attaining 4·10^5^ CFU/mL compared to its single culture counterpart. By the third day, during the lag phase, the population difference reached its peak, with nearly a 2-log reduction in B1 cells in the co-culture. At the end of the fermentation period, the final population of B1 in the co-culture was significantly lower at 1.09·10^7^ ± 0.08·10^7^ CFU/mL, compared to 3·10^8^ ± 0.11·10^8^ CFU/mL in the single culture (*p* < 0.05), demonstrating a strong antagonistic activity under the tested conditions. These findings indicate that *Metschnikowia pulcherrima* Mp2, while exerting a strong inhibitory effect on the growth of *Brettanomyces bruxellensis* B1, did not completely eliminate the spoilage yeast. This suggests that Mp2’s bioprotective activity is restricted to growth limitation rather than killing activity under these conditions. Here, again we can exclude competition for nutrients, since growth inhibition of *Brettanomyces bruxellensis* occurred very soon during the co-culture (24 h). Additionally, acetic acid, a key oenological metabolite associated with undesirable vinegar-like off-flavors at high concentrations [62] was analyzed (Figure 2A,B). In the co-culture with *Hanseniaspora uvarum* Hu3137 with Mp2, a very low amount of acetic acid was present, with levels not exceeding 0.04 g/L, comparable to its single culture profile. However, when co-inoculated with *Brettanomyces bruxellensis* strains B1 and B250, the acetic acid concentration was 0.6 g/L (Figure 2A,B), as these spoilage yeasts are well known for acetic acid production, which negatively impacts wine sensory quality [63]. These results confirm that neither L-lactic acid nor acetic acid produced by Mp2 contributed to the inhibition of *Hanseniaspora uvarum* Hu3137 or *Brettanomyces bruxellensis* B1 and B250, thereby excluding their role as primary mechanisms of bioprotection. This conclusion is further supported by previous field trials, which demonstrated that while the presence of acetic acid negatively affected *Brettanomyces bruxellensis* growth under aerobic conditions, it had no significant impact under oxygen-limited environments—consistent with our experimental conditions under semi-anaerobic conditions [64]. The production of metabolites and/or pulcherriminic acid was not involved either, as explained above. Our results suggest that another mechanism was involved in the *Brettanomyces bruxellensis* inhibition observed. To monitor the effect of ethanol and L-lactic acid on spoilage yeasts, we introduced these two compounds directly into the medium (Figure 4).

Among the *Lachancea thermotolerans* strains tested, Lt45 exhibited a growth profile and inhibitory behavior similar to *Metschnikowia pulcherrima* Mp2 against the implantation of *Brettanomyces bruxellensis* B1 at 22 °C. In contrast, Lt29 demonstrated a more pronounced inhibitory effect, as evidenced by a lower population of cells and maximum specific growth rate (μmax) for B1 in the co-culture (*p* < 0.05) (Figure 2A and Table 4). During single culture fermentation (Figure 2A), B1 increased its cell density from an initial concentration of 1.15.10^4^ CFU/mL to a maximum of 1.10^8^ CFU/mL at day 3, stabilizing at approximately 7.5·10^7^ ± 0.5·10^7^ CFU/mL (μmax = 0.063 h⁻^1^ ± 0.001) until the end of fermentation (day 9). However, in the co-culture, *Brettanomyces bruxellensis* grew from 1.18.10^4^ CFU/mL to 2·10^5^ CFU/mL within the first 3 days but subsequently exhibited a reduced growth rate, reaching a final concentration of 3.25·10^6^ ± 0.16·10^6^ CFU/mL (μmax = 0.03 h⁻^1^ ± 0.003). Despite the inhibitory effects observed, *Brettanomyces bruxellensis* maintained its culturability throughout the 9-day fermentation period.

The antagonistic effect of *Metschnikowia pulcherrima* Mp2 and *Lachancea thermotolerans* strains Lt29 and Lt45 against *Brettanomyces bruxellensis* B250 strain demonstrated a growth inhibition trajectory comparable to that observed with the B1 strain. However, the sensitivity of the B250 strain was notably higher, as evidenced by a reduction in its cell density, with nearly a half-log difference in inhibition compared to B1 (*p* < 0.05). This suggests that the B250 strain was more susceptible to the inhibitory actions of these bioprotectant yeasts under the conditions tested. Thus, the hypothesis of these results was the existence of the strain-specific response of *Brettanomyces bruxellensis* to different bioprotectant yeasts, which aligned with previous studies (such as *Torulaspora delbrueckii*, *Komagataella phaffii*, *Pichia anomala*, *Wickerhamomyces anomalus*, and *Kluyveromyces wickerhamii*) [26,35,40,41], emphasizing the importance of evaluating multiple spoilage yeast strains to fully understand the spectrum of inhibition.

With an initial concentration of *Hanseniaspora uvarum* and *Brettanomyces bruxellensis* at 5·10^4^ CFU/mL, *Metschnikowia pulcherrima* Mp2 exhibited a strong bioprotective effect, though its efficacy varied between the two spoilage yeasts. Therefore, *Metschnikowia pulcherrima* was shown to thrive in mixed cultures without experiencing microbial inhibition, reinforcing its suitability as a bioprotective agent in fermentation processes.

When analyzing the growth inhibition curve of B1 and B250 by Mp2, it is clear that the inhibition mechanism is not linked to the presence of either lactic acid, ethanol, or acetic acid, since when inhibition started (day 1–2) the concentrations of these metabolites were very low. Iron chelation did not appear to play a role in the mechanism observed, as the SM utilized contained no trace amounts of iron. Consequently, the inhibition observed is likely attributable to an alternative biochemical or molecular mechanism.

Regarding the inhibition of *Brettanomyces bruxellensis* by *Lachancea thermotolerans*, while inhibition by Lt45 is not linked to the metabolites analyzed, for Lt29, these metabolites might have played a role since they were produced in a significant amount. In order to investigate whether these metabolites were involved in the inhibition mechanism, B250 and B1 growths in the presence of ethanol and lactic acid (Figure 4A,B) were monitored. It appeared that the concentration tested corresponding to those found in the co-culture did not lead to growth inhibition, demonstrating that inhibition is not related to the production of these metabolites. A study by Fernández et al. [65] showed that the killing activity of killer toxin CF20KT produced by *Wickerhamomyces anomalus* CF20 and its cell-free supernatant (CFS) was the main contributor to the inhibition mechanism against six pathogenic strains and one strain of *Candida* spp. This inhibition was mainly caused by the secreted killer toxins with smaller contributions from the volatile compounds acetic acid and ethyl acetate. Moreover, the increased production of medium-chain fatty acid (MCFA) ethyl esters during mixed fermentation with *Saccharomyces cerevisiae* has been linked to elevated MCFA levels induced by the presence of *Hanseniaspora uvarum* Yun268 [66]. The authors suggested that *Hanseniaspora uvarum* not only tolerates but may also promote environments with higher MCFA concentrations. Therefore, most medium-chain fatty acids probably do not act as antagonistic compounds against *Hanseniaspora uvarum* in winemaking contexts.

### 3.4. Assessment of Killer Toxin Activity on Agar

**Figure 5:**
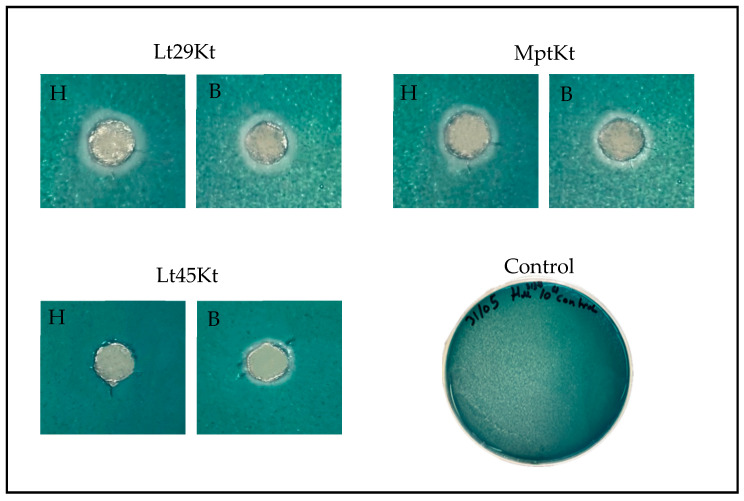
Well plate assay of semi-purified toxins Lt29Kt, Lt45Kt, and Mp2Kt. *B. bruxellensis* (B1) and *H. uvarum* (Hu3137) of initial concentration 10_4_ CFU/mL were used as sensitive strains spread on YPD-MB pH 3.5 medium. B: B1; H: Hu3137. Experiment was conducted in triplicate.

Following production (300 mL) in synthetic must of pH 3.5 and subsequent ethanol purification, the production of native killer toxins—Mp2Kt, Lt29Kt, and Lt45Kt—was confirmed after 48 h of growth. The killing ability of the produced toxins was examined to determine whether the bioprotective yeast’s activity on sensitive strains was due to the production of protein-based killer toxins, i.e., those previously characterized in various yeast species across different agar media. A well-diffusion method in YPD-BM media buffered to pH 3.5 was used. Wells were infused with Mp2Kt, Lt29Kt, and Lt45Kt and then inoculated with initial concentrations of 10^4^ CFU/mL of the sensitive strains Hu3137, B1, and B250. The sizes of the inhibition halos, which correspond to increasing concentrations of each killer toxin, are documented in Table 5 and Figure 5.

In vitro assays demonstrated that Lt29Kt exhibited the highest inhibitory activity among the toxins tested, effectively inhibiting *Hanseniaspora uvarum* Hu3137 and both *Brettanomyces bruxellensis* B1 and B250 strains, even at the minimal concentration of 0.185 mg/mL (Table 5). Lt45Kt, also derived from *Lachancea thermotolerans*, displayed selective activity, showing minimal effects on *Brettanomyces bruxellensis* strains B1 and B250 and no inhibitory effect on Hu3137, indicating a limited inhibitory range compared to Lt29Kt. Concerning Mp2Kt, it exerted a moderate inhibitory effect on these spoilage yeasts, evidenced by smaller halos, as shown in Table 5 and Figure 5. Notably, Lt29Kt, Lt45Kt, and Mp2Kt displayed a dose-dependent increase in antimicrobial efficacy, as reflected by progressively larger inhibition halos with increasing concentrations, as also reported by [26,31,37].

These results confirm the inhibitory effects of these killer toxins and support the hypothesis that they represent one of the mechanisms used by the bioprotective yeasts *Lachancea thermotolerans* and *Metschnikowia pulcherrima* to inhibit *Hanseniaspora uvarum* and *Brettanomyces bruxellensis*.

### 3.5. Inhibitory Effect of Killer Toxins on Spoilage Yeasts in Synthetic Must

Killer toxins were extracted after 2 days of growth corresponding mainly to the step where inhibition was first observed.

The extracted toxins Mp2Kt, Lt29Kt, and Lt45Kt were added in a synthetic must at pH 3.5, at concentrations of 0.185 and 0.500 mg/L. The highest concentration was approximately equivalent to the corresponding cell population present during the co-culture experiments.

**Figure 6:**
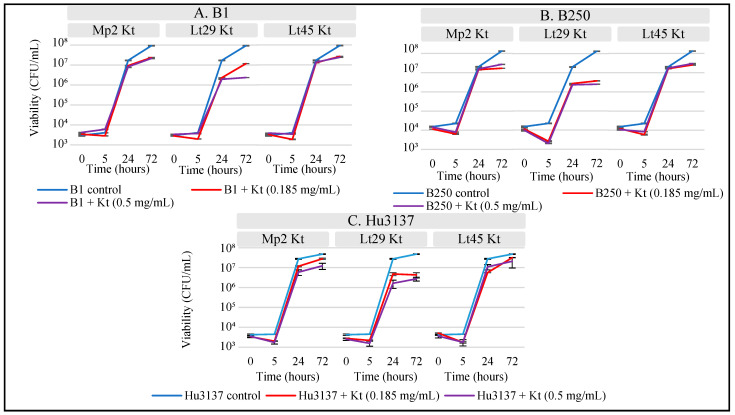
Viable cell counts of *Hanseniaspora uvarum* and *Brettanomyces bruxellensis* after 72 h of incubation with different concentrations of killer toxins. (**A**–**C**) graphs represent sensitive yeast incubated with Mp2Kt, Lt29Kt, and Lt45 Kt. (**A**) B1, (**B**) B250, (**C**) Hu3137. (**—**) Sensitive control where no toxin was added; (**—**) Kt 0.185 mg/mL; (**—**) Kt 0.5 mg/mL. Cell population determination is reported as mean value ± standard deviations (error bars) of 3 replications.

*Hanseniaspora uvarum* (*Hu*3137) and *Brettanomyces bruxellensis* (*B*1 *and B*250) were inoculated at an initial concentration of 10^4^ CFU/mL. The effect of the killer toxins on yeast viability was monitored at time points 0, 5, 24, and 72 h post-toxin addition, through viable plate counts. Figure 6 displays the growth curves of viable *Hanseniaspora uvarum* and *Brettanomyces bruxellensis* cells during growth in synthetic must.

Initial treatments with 0.185 mg/mL of Lt29Kt resulted in a marked inhibition of Hu3137, reducing cell numbers to 4.80·10^6^ CFU/mL after 24 h, underlining a significant decrease compared to the control, which maintained a population of 2.75·10^7^ CFU/mL (Figure 6C). Increasing the concentration of Lt29Kt to 0.500 mg/mL enhanced the inhibitory effect, reaching a reduction in viability exceeding 1 logarithmic unit (*p* < 0.05). This suppression was sustained for 72 h, with treated populations stabilizing at approximately 4.70·10^6^ and 4.30·10^6^ CFU/mL for 0.185 mg/mL and 0.500 mg/mL concentrations, respectively, while the control population for Hu3137 grew to 4.76·10^7^ CFU/mL. In tests against *Brettanomyces bruxellensis* strains B1 and B250, the application of 0.500 mg/mL of Lt29Kt initiated a substantial reduction in yeast growth within 24 h, which persisted, maintaining a 1.5 logarithmic decrease compared to the control population at 72 h (Figure 4A,B). At a lower concentration of 0.185 mg/mL, Lt29Kt exerted a similar level of inhibition on B250 but was less effective against B1, illustrating a dose-dependent variability in response among the different yeast strains. Lt29Kt demonstrates strong bioprotective efficiency, capable of inhibiting the growth of spoilage yeasts commonly encountered in winemaking. The concentration-dependent effects observed indicate that higher doses of the toxin were more effective for controlling yeast viability. The toxin’s impact on *Hanseniaspora uvarum* viability showed variable effects, suggesting that different yeast species may exhibit different levels of sensitivity to the same killer toxin.

At a concentration of 0.500 mg/mL, Lt45Kt and Mp2Kt effectively inhibited the growth of *Hanseniaspora uvarum* (Hu3137) by approximately 0.7 and 0.5 log units, respectively, during fermentation, and an almost similar reduction at a lower concentration of 0.185 mg/mL was also maintained. Interestingly, while Hu3137 resumed normal growth after 72 h at the lower concentration, the antimicrobial effect persisted at the higher concentration. Interestingly, both concentrations of Lt45Kt and Mp2Kt effectively reduced the growth of *Brettanomyces bruxellensis* strains B1 and B250, with reductions exceeding 0.5 log order after 72 h of inoculation (Figure 6A,B). This consistent effect of Lt45Kt aligns with findings from [31] that not all killer toxins behave in a dose-dependent manner. While toxins such as D2 and D15 extracted from *Wickerhamomyces anomalus* DiSVA2 and *K. wickerhamii* DiSVA15, respectively, showed increased effectiveness up to lower concentrations (0.1 mg/mL and 0.05 mg/mL), the D28 toxin from *Wickerhamomyces anomalus* DiSVA671 only significantly inhibited yeast at a much higher concentration of 1 mg/mL, highlighting variability in the dose–response among different killer toxins.

The results obtained by adding killer toxin in the fermentation medium did not correspond exactly to what was observed in the co-culture, despite the fact that the concentration of killer toxin added corresponded theoretically to the population of bioprotectant present in the co-culture. However, it is possible that a larger amount of secreted toxins was present in the co-culture. Indeed, when toxins are extracted, a yield of 100% cannot be obtained during the production and purification protocol [38]. The studies of Comitini et al. [41,67] highlighted a yield of only 70 and 15.2% from partially purified crude extract and the second step of ultrafiltration, respectively. In our experiment, however, only a fraction of these toxins were isolated and tested against *Hanseniaspora uvarum* and *Brettanomyces bruxellensis*, in contrast to other studies where a yield of 12.3–55.9 g/L was obtained, a value that is much higher than that secreted from a normal concentration of yeasts [31]. Despite the limited quantity of toxins we used, these were still effective in substantially inhibiting the growth of the spoilage yeasts. Another hypothesis is that toxins generated in the natural context of the co-culture might display greater efficacy than their purified equivalents. Such discrepancies could elucidate the varied inhibitory effects noted between co-culture experiments and those employing purified toxins, indicating that the environmental context and production protocol of toxins critically affect their inhibitory capabilities. During the extraction process, the toxins were exposed to high concentrations of ethanol, lower temperatures, and several steps of centrifugation, which could have altered their activity. This finding underscores the potency of killer toxins as a mechanism employed by bioprotective yeasts to suppress spoilage yeast activity, particularly in enological conditions.

In this study, Lt29Kt, derived from *Lachancea thermotolerans*, was demonstrated for the first time to be active on *Hanseniaspora uvarum* and *Brettanomyces bruxellensis* spoilage yeasts in synthetic must under conditions that simulate winemaking conditions. While the toxins Mp2Kt and Lt45Kt also showed inhibitory effects on these yeasts, their efficacy was notably lower than that of Lt29Kt. This disparity in inhibitory potency suggests that higher concentrations of Mp2Kt and Lt45Kt may be necessary to achieve comparable levels of yeast inhibition. These findings underline the importance of optimizing killer toxin concentrations to maximize efficacy while ensuring the quality and safety of wine. They can safeguard against spoilage without disrupting fermentation by *Saccharomyces cerevisiae*, the key fermentation yeast. This experiment also demonstrated that while cell–cell contact or quorum sensing could not be excluded as mechanisms of bioprotection, our results clearly demonstrate that some killer toxins play a major role in bioprotection mechanisms.

## 4. Conclusions

Research has shown that certain non-*Saccharomyces* species exhibit killer activity against apiculate yeasts (*Hanseniaspora uvarum*) and various *Brettanomyces bruxellensis* strains [31,33,40,41,54,67]. In our study, experiments involving *Metschnikowia pulcherrima* and *Lachancea thermotolerans* were carefully designed to evaluate their potential as bioprotectants. These investigations were structured around three primary objectives: (i) assessing the extent of killer activity these yeasts exhibit against *Hanseniaspora uvarum* and *Brettanomyces bruxellensis* in synthetic must; (ii) investigating the role of metabolites in their antimicrobial mechanisms; and (iii) evaluating the performance of semi-purified killer toxins in conditions simulating those found in winemaking.

All three of the bioprotective *Lachancea thermotolerans* and *Metschnikowia pulcherrima* strains selected showed either significant killing activity or an inhibitory effect on the growth of *Hanseniaspora uvarum* and *Brettanomyces bruxellensis* yeasts in our experimental conditions.

These results demonstrated that the inhibition of the growth of the two species *Hanseniaspora uvarum* and *Brettanomyces bruxellensis* is not linked to the production of metabolites. Furthermore, concerning *Metschnikowia pulcherrima*, in our experimental conditions, it appeared that the inhibition mechanism was not due to the chelation of iron by pulcherriminic acid. On the other hand, our results showed that killer toxins are particularly involved in bioprotection mechanisms. To optimize must bioprotection, these results suggest that the killer activity of the strains against spoilage microorganisms in oenological conditions should be taken into account in the selection criteria of bioprotective strains.

## Figures and Tables

**Table 1:** Initial cell concentration (measured in CFU/mL) employed for single cultures and co-cultures of bioprotectant (*Lachancea thermotolerans* and *Metschnikowia pulcherrima*) strains and sensitive strains (*Hanseniaspora uvarum* and *Brettanomyces bruxellensis*) mix.

	Single Control Culture (CFU/mL)	Co-Culture Mix (CFU/mL)	
		Sensitive Strains	Bioprotectant Strains
*Brettanomyces bruxellensis* single culture (B1 or B250)	1 × 10^4^	-	
*Hanseniaspora uvarum* single culture (Hu3137)	1 × 10^4^	-	
*Lachancea thermotolerans* single culture (Lt29 or Lt45)	1 × 10^6^	-	
*Metschnikowia pulcherrima* single culture (Mp2)	1 × 10^6^	-	
Co-culture of *bioprotectant* × sensitive strains	-	1 × 10^4^	1 × 10^6^

**Table 2:** List of killer *non-Saccharomyces* strains and their inhibition potentiality along with the source from which they were derived. Nd: activity not determined.

Killer Yeast Codes	Killer Genera	Isolation Source	Killer Activity Agar Medium				
			B1	B250	B3	B7	Hu3137
Sb 36340	*Starmerella bacillaris*	Carignan	++	+	+	+/−	Nd
Sb 36341	*Starmerella bacillaris*	Tempranillo	+/−	+	+	−	Nd
Lt 28606	*Lachancea thermotolerans*	Karech Noir Must	+	+	+	+/−	Nd
Lt 28607	*Lachancea thermotolerans*	Karech Noir Must	+	+	++	+/−	Nd
Lt 28615	*Lachancea thermotolerans*	Karech Noir Must	+/−	+	+	++	Nd
Lt 28645	*Lachancea thermotolerans*	Karech Noir Must	+	++	+	++	+
Lt 28655	*Lachancea thermotolerans*	Karech Noir Must	+	++	+	++	Nd
Lt 29126	*Lachancea thermotolerans*	Grenache Must	+	+/−	+	+	Nd
Lt 29128	*Lachancea thermotolerans*	Grenache Must	++	++	+	++	Nd
Lt 29129	*Lachancea thermotolerans*	Grenache Must	++	++	++	++	++
Lt 29130	*Lachancea thermotolerans*	Grenache Must	++	++	++	++	Nd
Lt 29134	*Lachancea thermotolerans*	Grenache Must	++	++	+/−	+	Nd
Lt 29136	*Lachancea thermotolerans*	Grenache Must	+/−	+/−	+/−	+/−	Nd
Lt 29139	*Lachancea thermotolerans*	Grenache Must	+/−	+/−	++	++	Nd
Lt 29140	*Lachancea thermotolerans*	Grenache Must	++	++	+/−	+/−	Nd
Lt 29143	*Lachancea thermotolerans*	Grenache Must	++	++	++	++	Nd
Lt 29147	*Lachancea thermotolerans*	Grenache Must	++	++	++	++	Nd
Lt 29694	*Pichia kudriavzevii*	Mawardi Rouge Must	−	−	+/−	+/−	Nd
Lt 29698	*Pichia kudriavzevii*	Mawardi Rouge Must	−	−	+/−	+/−	Nd
Lt 29702	*Pichia kudriavzevii*	Mawardi Rouge Must	−	−	+/−	+/−	Nd
Mp2	*Metschnikowia pulcherrimin*	Commercial	+	++	++	+	++

**Table 3:** Killing activity of non-Saccharomyces strains versus Saccharomyces strains.

Selected Bioprotectant Strains	Target *S. cerevisiae* Strains			
	S342	S340	S334	VL2
Mp2	K− R+	K− R+	K− R+	Nd
Lt29	K− R+	K− R+	K− R+	K− R+
Lt45	K− R+	K− R+	K+ R+	K+ R+

Killing activity against *S. cerevisiae* “K+”; no killing activity against *S. cerevisiae* “K−”. Resistances of bioprotectant strains to *S. cerevisiae* “R+”; sensitivity of bioprotectant to *S. cerevisiae* “R+”. Incubated at 22 °C, pH 3.5, and same initial viability of (10^6^ CFU/mL).

**Table 4:** Growth parameters of *Brettanomyces bruxellensis* (B1 and B250) and *Hanseniaspora uvarum* (Hu3137) comparison in single culture and co-culture in presence of each of *Metschnikowia pulcherrima* (Mp2) and *Lachancea thermotolerans* (Lt29 andLt45) strains, incubated in synthetic must of 3.5 pH at 22 °C.

**Sensitive Strain**	**Killer Strain ^2^**	**μ max** **(h^−1^)**	**Final Population** **(CFU/mL) (9 Days)**
B1 ^1^		0.126 ± 0.001 ^a,3^	2.72·10^8^ ± 0.11·10^8 a^
	Mp2	0.058 ± 0.003 ^c^	1.09·10^7^ ± 0.08·10^7 c^
	Lt29	0.044 ± 0.001 ^b^	8.93·10^6^ ± 0.94·10^6 c^
	Lt45	0.065 ± 0.001 ^c^	1.86·10^7^ ± 0.14·10^7 b^
**Sensitive Strain**	**Killer Strain ^2^**	**μ max** **(h^−1^)**	**Final Population** **(CFU/mL) (9 Days)**
B250 ^1^		0.123 ± 0.002 ^a,3^	2.7·10^8^ ± 0.06·10^8 a^
	Mp2	0.034 ± 0.002 ^c^	1.15·10^7^ ± 0.16·10^7 c^
	Lt29	0.045 ± 0.001 ^c^	2.07·10^6^ ± 0.41·10^6 b^
	Lt45	0.073 ± 0.004 ^b^	1.54·10^7^ ± 0.24·10^7^
**Sensitive Strain**	**Killer Strain ^2^**	**μ max** **(h^−1^)**	**Final Population** **(CFU/mL) (9 Days)**
Hu3137 ^1^		0.204 ± 0.001 ^a,3^	5.8·10^7^ ± 1.2·10^7 a^
	Mp2	0.03 ± 0.002 ^b,d^	2.64·10^4^ ± 1.94·10^4 b^
	Lt29	0.03 ± 0.003 ^b^	0 ^b^
	Lt45	0.053 ± 0.004 ^d^	0 ^b^

^1^ Initial concentration 10^4^ CFU/mL, ^2^ initial concentration 10^6^ CFU/mL. ^3^ Letter corresponds to statistical groups (post hoc “Bonferroni”, *p* < 0.05) obtained by the pairwise comparison of values between B1, B250, and Hu3137 single culture and co-culture with the bioprotectant strains at initial concentrations of 10^4^ and 10^6^ CFU/mL, respectively.

**Table 5:** Inhibition zone diameters of sensitive *Hanseniaspora uvarum* and *Brettanomyces bruxellensis*. The data show the zones of growth inhibition of the sensitive yeast strains when exposed to various concentrations “0.500 and 0.185 mg/mL” of each killer toxin immediately following their extraction.

Killer Toxins	Halo Diameter (mm)					
	*H. uvarum* “Hu3137”		*B. bruxellensis* “B1”		*B. bruxellensis* “B250”	
	0.500 mg/mL	0.185 mg/mL	0.500 mg/mL	0.185 mg/mL	0.500 mg/mL	0.185 mg/mL
Lt29Kt	10	7	9	5	8	5
Lt45Kt	0	0	4	1	4	2
Mp2Kt	6	0	5	3	5	2

## Data Availability

The original contributions presented in the study are included in the article, further inquiries can be directed to the corresponding author.
